# Low Glucose but Not Galactose Enhances Oxidative Mitochondrial Metabolism in C2C12 Myoblasts and Myotubes

**DOI:** 10.1371/journal.pone.0070772

**Published:** 2013-08-05

**Authors:** Moustafa Elkalaf, Michal Anděl, Jan Trnka

**Affiliations:** 1 Laboratory for Metabolism and Bioenergetics, Third Faculty of Medicine, Charles University, Prague, Czech Republic; 2 Centre for Research on Diabetes, Metabolism and Nutrition, Third Faculty of Medicine, Charles University, Prague, Czech Republic; Laurentian University, Canada

## Abstract

**Background:**

Substituting galactose for glucose in cell culture media has been suggested to enhance mitochondrial metabolism in a variety of cell lines. We studied the effects of carbohydrate availability on growth, differentiation and metabolism of C2C12 myoblasts and myotubes.

**Methodology/Principal Findings:**

We measured growth rates, ability to differentiate, citrate synthase and respiratory chain activities and several parameters of mitochondrial respiration in C2C12 cells grown in media with varying carbohydrate availability (5 g/l glucose, 1 g/l glucose, 1 g/l galactose, and no added carbohydrates). C2C12 myoblasts grow more slowly without glucose irrespective of the presence of galactose, which is not consumed by the cells, and they fail to differentiate without glucose in the medium. Cells grown in a no-glucose medium (with or without galactose) have lower maximal respiration and spare respiratory capacity than cells grown in the presence of glucose. However, increasing glucose concentration above physiological levels decreases the achievable maximal respiration. C2C12 myotubes differentiated at a high glucose concentration showed higher dependency on oxidative respiration under basal conditions but had lower maximal and spare respiratory capacity when compared to cells differentiated under low glucose condition. Citrate synthase activity or mitochondrial yield were not significantly affected by changes in the available substrate concentration but a trend towards a higher respiratory chain activity was observed at reduced glucose levels.

**Conclusions/Significance:**

Our results show that using galactose to increase oxidative metabolism may not be applicable to every cell line, and the changes in mitochondrial respiratory parameters associated with treating cells with galactose are mainly due to glucose deprivation. Moderate concentrations of glucose (1 g/l) in a growth medium are optimal for mitochondrial respiration in C2C12 cell line while supraphysiological concentrations of glucose cause mitochondrial dysfunction in C2C12 myoblasts and myotubes.

## Introduction

Skeletal muscle is an important tissue for energy metabolism in humans [1]–[4]. C2C12 murine myoblastic cell line is widely used as an *in vitro* model of skeletal muscle especially thanks to its capability to differentiate into muscle-like myotubes [5]–[7]. The composition of growth media is usually considered as a background condition, which has no significant effect on observed phenomena but many studies suggest that differences in glucose concentrations in media may have significant effects on the metabolic phenotype of model cell lines, in particular C2C12 murine myoblasts and myotubes.

Cells cultured in media with standard concentrations of glucose tend to acquire highly glycolytic phenotypes (“Crabtree effect”, [8]), which makes them less suitable as models for *in vitro* metabolic studies. Attempts have been made to overcome this phenomenon, by substituting glucose for galactose, which does not support anaerobic glycolysis. This is usually explained by the fact that galactose cannot be oxidised to pyruvate without prior conversion to glucose, which consumes two molecules of ATP, thus making anaerobic glycolysis useless as a source of energy. Galactose-fed cells then should rely on mitochondrial oxidative phosphorylation to produce ATP, hence providing us with a better model for studying mitochondrial function. Several studies have shown substantial changes in energy metabolism under such conditions and galactose-based media are often recommended to circumvent the Crabtree effect [9].

Since skeletal muscle lacks the ability to metabolise galactose [10], [11], a recent study suggested decreasing glucose concentration in growth media and investigated the effect of two glucose concentrations 5 mM and 25 mM on the basal and maximal respiration of differentiated C2C12 myotubes [12] and stated that cells grown and differentiated in high glucose environment possessed lower maximal respiratory capacity than those grown and differentiated in lower glucose level. A later study [13] found significant effects of a galactose medium on the metabolic function of human primary myoblasts. However, the authors compared cellular respiration in media with different compositions thus making it difficult to distinguish acute effects of substrate availability from longer-term phenotypic changes in cells grown in galactose-containing medium.

As our understanding of the effects of low glucose and galactose on cultured skeletal muscle cells is still far from satisfactory, in this paper we present a detailed study of changes of growth patterns and several parameters of mitochondrial metabolism in C2C12 myoblasts and myotubes in response to differing availability of glucose or galactose while trying to avoid some of the shortcomings of previously published work and provide experimental data showing all aspects of the relationship of cell culture conditions and metabolic activity.

## Results

### C2C12 do not Substitute Glucose with Galactose as Energy Substrate

**Figure 1:**
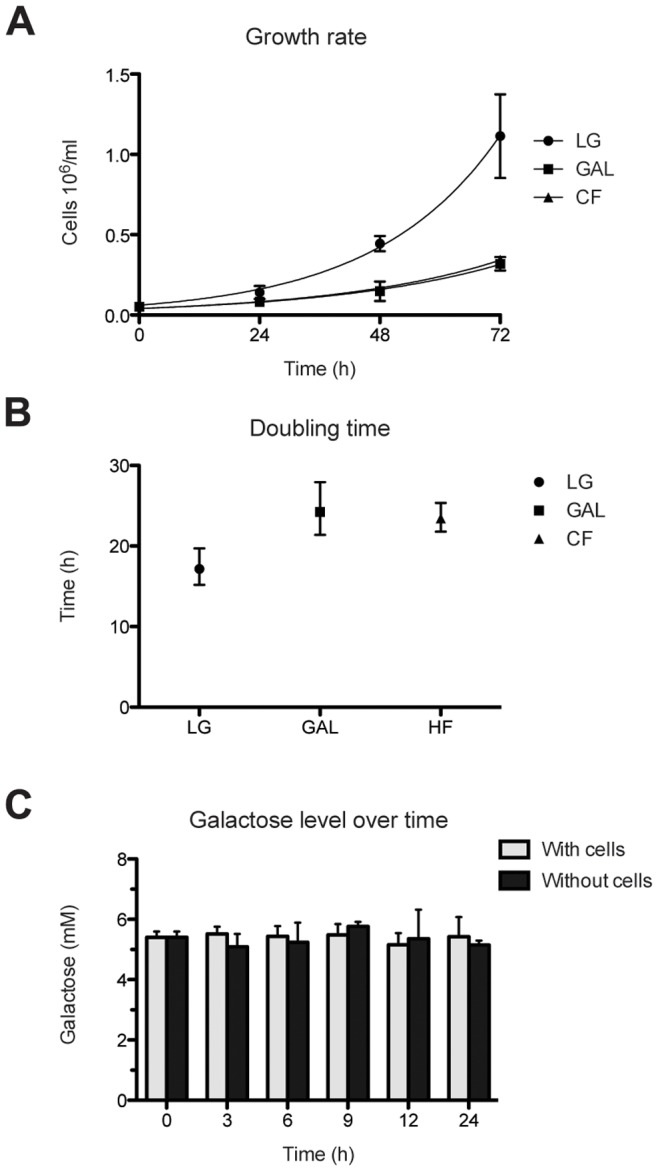
C2C12 cells grow more slowly without glucose and fail to consume galactose. **A.** Growth rate over 3 days. LG, 1 g/l glucose; GAL, 1 g/l galactose, CF, carbohydrate-free. **B.** Doubling time. **C.** Changes in galactose concentration in growth media over time. All results are presented as means and 95% CI, (n = 3, each experiment was performed in triplicate).

The comparison of growth rates of C2C12 cells grown in a medium supplemented with 1 g/l glucose (LG), 1 g/l galactose (GAL) or a medium with no added carbohydrates (CF), shows significantly faster growth in LG (doubling time 17.16 h, 95% CI[15.19, 19.70]*) compared with glucose-deficient conditions GAL (24.24 h, 95% CI[21.40, 27.93]) and CF (23.44 h, 95% CI[21.79, 25.36]) (Fig. 1A,B).

Based on these results we hypothesised that C2C12 cells are unable to use galactose as a major energy source and therefore we measured the consumption of galactose under these growth conditions. Our data show no significant change in galactose concentration in the medium over time in cell-containing wells compared to wells without cells (Fig. 1C).

### C2C12 Myoblasts Fail to Differentiate without Glucose

**Figure 2:**
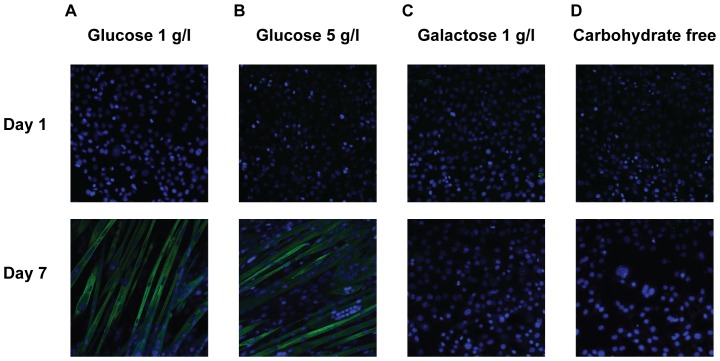
Cells lacking glucose fail to differentiate. Confluent C2C12 myoblasts were exposed to low-serum medium to differentiate into myotubes. **A, B.** Cells in low-glucose (1 g/l) as well as in high glucose (5 g/l) media fused to form multinucleated myotubes after 7 days in a differentiation medium and expressed myosin heavy chain. **C, D.** In galactose (5 g/l) and carbohydrate free media cells failed to differentiate after 7 days.

The ability to differentiate into myotubes is an important characteristic of C2C12 cells as a model of skeletal muscle. The differentiation process is associated with the expression of differentiation markers such as myosin heavy chain (MHC), which is considered a typical marker for terminally differentiated muscle cells [14]. Glucose starvation is known to impair the differentiation process [15], [16], therefore we investigated whether galactose can be substituted for glucose. Immediately after the induction of differentiation, cells did not express myosin heavy chain but after 7 days of differentiation we observed well formed MHC-positive multinucleated myotubes (Fig. 2A,B) in both LG and HG phenotypes. When glucose was not available we observed a major impairment of cell fusion and MHC expression and galactose was not sufficient to replace glucose (Fig. 2C,D).

### Changes in Glucose availability Affect Maximum and Spare Mitochondrial Respiratory Capacities in C2C12 Myoblasts

To identify changes in mitochondrial parameters due to growth conditions, we measured mitochondrial respiration of cells grown for 7 days in a medium with various concentrations of carbohydrates: HG (5 g/l glucose), LG (1 g/l glucose), GAL (1 g/l galactose, 0 g/l glucose) or CF (carbohydrate-free, 0 g/l of added sugars). Respiration of these cells was then measured under three conditions: A) in assay media containing the same carbohydrate as the growth medium for each phenotype, B) in a common assay medium with 1 g/l glucose and C) in a common assay medium with no glucose.

This allowed us to determine the changes to mitochondrial respiration under different substrate availability with experiment A being essentially a replication of the study by Aguer et al. 2011 [13] but using murine instead of human myoblasts. In addition to basal respiration we determined other mitochondrial parameters: basal mitochondrial respiration (basal cellular respiration minus non-mitochondrial respiration), ATP turnover-driven respiration (basal respiration minus oligomycin-inhibited respiration), maximal uncoupled respiration, spare respiratory capacity (maximal uncoupled respiration minus basal respiration), proton leak (oligomycin-inhibited respiration minus antimycin/rotenone-inhibited respiration) and non-mitochondrial respiration (antimycin/rotenone-inhibited respiration). All values in this section are in pmol(O_2_).min^−1^.(µg protein) ^−1^ with 95% CI in square brackets.

A. Under assay conditions identical to culture conditions we found marked differences in maximum and spare respiratory capacities (Table 1). A presence of glucose in either concentration led to an increase in maximal respiration (difference of means LG vs CF 16.13 [6.37, 25.89]*, LG vs GAL 14.14 [4.38, 23.90]*, HG vs CF 9.16 [−0.60, 18.92], HG vs GAL 7.17 [−2.59, 16.93]) and spare respiratory capacity (difference of means LG vs CF 15.7 [6.34, 25.06]*, LG vs GAL 14.35 [4.99, 23.70]*, HG vs CF 9.73 [0.37, 19.09]*, HG vs GAL 8.38 [−0.98, 17.73]).

**Table 1:** Mitochondrial respiratory parameters in assay media containing growth medium substrates (pmol(O_2_).min^−1^.(µg protein)^−1^ ).

	LG	HG	GAL	CF
Basal respiration	9.78 [0.48,19.08]	8.39 [4.25,12.54]	10.20 [6.17,14.24]	9.18 [3.42,14.81]
Basal mitochondrial respiration	6.52 [−0.25,13.29]	5.52 [4.06,6.99]	6.73 [1.52,11.93]	6.09 [3.61,8.57]
ATP turnover-driven respiration	5.48 [−0.06,11.57]	4.60 [2.57,6.63]	6.15 [2.83,9.47]	5.13 [2.30,7.97]
Maximum respiratory capacity	24.17 [14.24,34.09]	17.20 [14.21,20.18]	10.03 [−4.08,24.13]	8.04 [1.91,14.17]
Spare respiratory capacity	17.65 [7.11,28.18]	11.67 [7.27,16.08]	3.30 [−7.77,14.37]	1.95 [−6.00,9.90]
H^+^ Leak	1.04 [−0.23,2.31]	0.92 [−0.60,2.45]	0.57 [−1.45,2.60]	0.96 [0.58,1.34]
Non-mitochondrial respiration	3.26 [−0.06,6.58]	2.87 [−0.45,6.19]	3.48 [1.31,5.65]	3.03 [−0.36,6.41]

Data are shown as means and 95% CI, n = 3.

Interestingly, a further increase of glucose concentration over 1 g/l in the long-term culture medium caused a relative decrease in maximal respiration (difference of means LG vs HG 6.97 [−2.79, 16.73]) and in the spare respiratory capacity (difference of means LG vs HG from 5.97 [−3.39, 15.33]).

Differences among the different phenotypes in basal mitochondrial respiration and other respiratory parameters were insignificant.

B. When assayed in a medium supplemented with 1 g/l glucose (Table 2) we also observed that cells deprived of glucose during growth exhibited a lower maximal respiration (difference of means LG vs CF 14.35 [10.14, 18.56]*, LG vs GAL 15.3 [11.09, 19.51]*, HG vs CF 5.06 [0.85, 9.27]*, HG vs GAL 6.01 [1.80, 10.22]*) and spare respiratory capacity (difference of means LG vs CF 12.14 [6.60, 17.68]*, LG vs GAL 12.92 [7.38, 18.46]*, HG vs CF 2.85 [−2.69, 8.39], HG vs GAL 3.63 [−1.92, 9.17]). There was also slightly higher basal mitochondrial respiration and ATP turnover-driven respiration in the two glucose phenotypes compared to glucose-deprived cells. Differences in other mitochondrial parameters were insignificant.

**Table 2:** Mitochondrial respiratory parameters in glucose 1 g/l assay medium (pmol(O_2_).min^−1^.(µg protein)^−1^).

	LG	HG	GAL	CF
Basal respiration	9.65 [0.45,18.85]	8.37 [5.49,11.26]	6.76 [5.25,8.26]	6.42 [4.73,8.12]
Basal mitochondrial respiration	6.16 [0.60,11.71]	6.16 [4.43,7.89]	3.78 [1.35,6.21]	3.94 [2.16,5.73]
ATP turnover-driven respiration	5.37 [−0.06,10.8]	5.00 [3.93,6.06]	3.02 [1.90,4.13]	3.08 [2.01,4.15]
Maximum respiratory capacity	24.86 [20.83,28.89]	15.57 [11.93,19.2]	9.56 [5.51,13.61]	10.51 [6.25,14.77]
Spare respiratory capacity	18.70 [9.15,28.25]	9.41 [6.65,12.17]	5.78 [3.67,7.89]	6.56 [3.83,9.3]
H^+^Leak	0.79 [−0.74,2.31]	1.16 [0.31,2.01]	0.76 [−1.03,2.55]	0.87 [−0.19,1.93]
Non-mitochondrial respiration	3.50 [−1.59,8.59]	2.22 [0.32,4.11]	2.98 [0.81,5.15]	2.48 [1.81,3.14]

Data are shown as means and 95% CI, n = 3.

Similar to experiment A, going over 1 g/l glucose in the culture medium caused an even larger relative decrease in maximal respiration (difference of means LG vs HG 9.29 [5.08, 13.50]*), and in spare respiratory capacity (difference of means LG vs HG 9.29 [3.75,14.83]*).

C. Finally we assayed cells in a glucose-free medium (Table 3). Differences among the four phenotypes *within* this experiment were negligible. However, a comparison of experiments B and C shows that in the sudden absence of glucose in the assay medium the two glucose phenotypes (LG, HG) exhibited a significantly lower maximal respiration and spare capacity compared to experiment B, with similar values for other mitochondrial parameters. The long-term glucose-deprived cells (GAL, CF), on the other hand, had similar values of maximal and spare respiration in the absence (experiment C) as in the presence of glucose (experiment B) but higher basal mitochondrial respiration and ATP turnover-driven respiration (compare Tables 2 and 3).

**Table 3:** Mitochondrial respiratory parameters in glucose-free assay medium (pmol(O_2_).min^−1^.(µg protein)^−1^).

	LG	HG	GAL	CF
Basal respiration	9.21 [−1.26,19.69]	7.83 [−0.31,15.97]	10.29 [4.97,15.60]	7.75 [−4.60,20.11]
Basal mitochondrial respiration	6.50 [−0.74,13.74]	5.75 [0.02,11.48]	7.09 [4.18,10.00]	5.42 [−4.36,15.19]
ATP turnover-driven respiration	5.48 [−1.05,12.02]	4.58 [0.05,9.11]	6.00 [3.93,8.07]	4.35 [−3.52,12.22]
Maximum respiratory capacity	11.76 [8.85,14.67]	7.80 [1.50,14.09]	11.09 [−4.48,26.66]	8.31 [−7.05,23.66]
Spare respiratory capacity	5.26 [−3.99,14.51]	2.05 [−0.58,4.67]	4.00 [−9.00,17.00]	2.89 [−5.92,11.70]
H^+^ Leak	1.02 [0.05,1.99]	1.17 [−0.04,2.38]	1.09 [−0.17,2.34]	1.07 [−0.91,3.05]
Non-mitochondrial respiration	2.71 [−0.85,6.27]	2.08 [−0.41,4.56]	3.20 [0.33,6.07]	2.33 [−0.64,5.31]

Data are shown as means and 95% CI, n = 3.

### Glucose Concentration during Differentiation Affects Mitochondrial Parameters in C2C12 Myotubes

Myotubes differentiated in a high glucose environment exhibited somewhat higher basal mitochondrial respiration when compared to LG myotubes (difference of means LG vs HG −3.58 [−6.32, −0.84]*) and ATP turnover-driven respiration (difference of means LG vs HG −2.68 [−4.37, −0.98]*). On the other hand, the LG phenotype maintained much higher maximal respiration (difference of means LG vs HG 16.07 [3.16, 28.98]*) and spare respiratory capacity (difference of means LG vs HG 19.65 [6.92, 32.38]*).

### Glycolytic Activity

We found no significant differences in basal and oligomycin-stimulated rate of extracellular acidification (ECAR) among the substrate phenotypes in myoblasts or myotubes, however, myoblasts exhibited significantly higher values for both parameters compared to myotubes (Table 5).

**Table 5:** Glycolytic activity (ECAR) in 1 g/l glucose environment (mpH.min^−1^.(µg protein)^−1^).

Phenotype	Basal	Oligomycin-stimulated	Glycolytic reserve
Myoblasts			
LG	3.27 [2.27,4.27]	4.60 [3.22,5.98]	1.33 [0.91,1.75]
HG	3.46 [1.57,5.34]	4.94 [1.66,8.22]	1.48 [0.07,2.89]
GAL	2.61 [1.10,4.12]	3.48 [0.71,6.26]	0.87 [−0.40,2.15]
CF	3.77 [0.46,7.07]	5.09 [0.58,9.59]	1.32 [0.09,2.55]
Myotubes			
LG	0.42 [0.26,0.59]	0.83 [0.46,1.20]	0.41 [−0.01,0.82]
HG	0.43 [0.22,0.63]	0.72 [0.04,1.41]	0.30 [−0.18,0.77]

Data are shown as means and 95% CI, n = 3.

### Mitochondrial Mass Markers Remain Unchanged Under Various Culture Conditions

We measured citrate synthase (CS) activity in whole cell lysates and mitochondrial yield (µg mitochondrial protein/µg total cellular protein) in all phenotypes as indicators of mitochondrial mass [17]–[19]. We found no significant differences in CS activity among the substrate phenotypes, however, myotubes had a significantly higher CS activity than myoblasts. Mitochondrial yield was similar in all myoblast phenotypes but LG myotubes had a higher mitochondrial content than HG myotubes (Table 6).

**Table 6:** Mitochondrial mass markers in myoblasts and myotubes.

Phenotype	CS	Mitochondrial yield
Myoblasts		
LG	1.73 [1.05,2.42]	0.12 [0.09,0.15]
HG	1.62 [1.25,2.00]	0.09 [0.07,0.10]
GAL	1.85 [1.21,2.49]	0.13 [0.09,0.16]
CF	1.55 [0.96,2.14]	0.09 [0.01,0.18]
Myotubes		
LG	2.94 [2.50,3.38]	0.18 [0.17,0.20]
HG	2.88 [2.52,3.24]	0.12 [0.05,0.19]

Citrate synthase activity is expressed as (nmol.min^−1^.(µg protein) ^−1^). Mitochondrial yield was determined as µg of mitochondrial protein content per µg of total cellular protein. Data are shown as means and 95% CI, n = 3.

### Respiratory Chain Activities Tend to Increase in the Absence of Glucose

The activity of all respiratory chain complexes tended to be higher in the glucose-starved phenotypes GAL and CF compared to LG and HG with a significant difference in complex IV activity (difference of means LG vs GAL −2.59 [−4.22, −0.97]*, LG vs CF −1.48 [−3.11, 1.39], HG vs GAL −3.03 [−4.65, −1.40]*, HG vs CF −1.92 [−3.54, −0.29]* µΔln(A_550_).min^−1^.( µg protein)^−1^). No significant differences were observed within these two groups (Table 7). In myotubes the LG phenotype exhibited significantly higher activities of complex I (difference of means LG vs HG 93.83 [41.18, 146.5]* pmol(DCIP).min^−1^.(µg protein)^−1^) and complex III (difference of means LG vs HG 41.27 [20.76, 61.78]* pmol(cytochrome *c*).min^−1^.(µg protein)^−1^).

**Table 7:** Respiratory chain enzymatic activity in myoblasts and myotubes.

	Complex I^†^	Complex II^†^	Complex III^¶^	Complex IV^§^
Myoblasts				
LG	56.70 [23.33,90.07]	21.89 [15.57,28.12]	32.29 [12.34,52.24]	3.31 [2.15,4.48]
HG	58.44 [17.19,99.69]	22.00 [11.40,32.59]	26.35 [−11.83,64.53]	2.88 [2.00,3.76]
GAL	84.78 [33.26,136.30]	27.39 [3.85,50.93]	54.94 [9.85,100.03]	5.91 [3.35,8.47]
CF	86.49 [69.58,103.40]	30.31 [4.48,56.14]	56.07 [−14.94,127.07]	4.80 [3.87,5.73]
Myotubes				
LG	176.72 [125.70,227.75]	45.25 [32.09,58.42]	64.67 [42.36,86.99]	6.42 [2.85,9.99]
HG	82.89 [19.22,146.55]	37.08 [22.98,51.18]	23.40 [0.75,46.05]	4.55 [2.88,6.21]

Activities were expressed as ^†^pmol(DCIP).min^−1^.(µg protein)^−1^, ^¶^pmol(cytochrome *c*).min^−1^.(µg protein)^−1^, ^§^µΔln(A_550_).min^−1^.(µg protein)^−1^.

Data are shown as means and 95% CI, n = 3.

## Discussion

Galactose has been used in many studies in attempts to abolish the Crabtree effect [9], [20], [21], and to increase the reliance on mitochondrial oxidative respiration in models used to study mitochondrial function/dysfunction. The present study shows that extending this model to some cell lines may be complicated. We investigated the effect of carbohydrate availability on the metabolic function of murine myoblast line C2C12. Previous studies on murine myoblasts showed, in general, that despite the availability of oxidisable fuels in the incubation medium, myoblasts in the early stage of differentiation derive approximately 60% of their energy demands by lactate production from glucose [22]. For that reason attempts to develop a more oxidative model were made either by lowering the glucose level [12] or by substituting galactose for glucose [13]. In the latter paper Aguer et al. presented data showing that the differentiation of human primary myoblasts in a galactose-containing medium reveals mitochondrial dysfunction in samples derived from formerly diabetic patients, who lost weight and became normoglycemic.

Our first finding was that growing myoblasts do not utilise available galactose in the medium and had a growth rate indistinguishable from cell grown in a carbohydrate-free environment. This matches with previous publications noting that the entry of galactose into the cells of isolated diaphragm muscle is limited, and galactose that enters the skeletal muscle remains essentially unmodified [10] and that myoblasts do not possess enzymes necessary for galactose metabolism [11]. We believe it is reasonable to assume that C2C12 cells in the absence of glucose rely on the metabolism of pyruvate and amino acids in the growth medium irrespective of the presence or absence of galactose.

A significant limitation of the galactose culture medium for C2C12 myoblasts is presented by our observation that they fail to differentiate in the absence of glucose, which is in good agreement with previous studies on glucose deprivation [15], [16]. Glucose starvation arrested the differentiation process and galactose in our experiments failed to provide a suitable substitute for cells, which then remained undifferentiated and some transformed into giant cells.

We also performed a detailed study of mitochondrial respiration of C2C12 cells grown in media with different amounts of glucose or galactose. Previous similar studies measured fewer parameters [12] or used different assay media for different phenotypes, which made direct comparison impossible [13]. We therefore used experimental designs which included analyses in the same assay medium for all phenotypes and further extended previous publications through performing experiments in different metabolic environments.

In an experimental setup directly comparable to Aguer et al. 2011 (Table 1), we observed markedly lower maximal and spare respiratory capacities of the GAL and CF phenotypes but no differences in basal or ATP turnover-driven mitochondrial respiration suggesting that starving C2C12 cells of glucose does not increase their oxidative metabolism. The disagreement between our results and the published study may be explained by hypothetical metabolic differences between primary human myoblasts and the C2C12 cell line.

In order to compare directly the metabolic phenotypes of cells grown in different media, we used a unified assay medium containing either 1 g/l glucose (Table 2) or no glucose (Table 3). When glucose was available in the assay environment, the no-glucose phenotypes exhibited lower basal and maximal respiration, which goes against the hypothesis that glucose-deprivation stimulates oxidative metabolism. This is further strengthened by the fact that the glucose phenotypes exhibited somewhat higher basal and ATP turnover-driven respiration suggesting a stronger reliance on oxidative metabolism under basal conditions. However, cells grown in high glucose had lower respiratory parameters than cells grown in low glucose, which suggests that an optimal respiratory phenotype of C2C12 cells is achieved at moderate glucose concentration. Interestingly, the difference between the two glucose phenotypes was only seen in the stimulated maximal respiration and spare respiratory capacity which points less to a generally glycolytic metabolism in the HG phenotype and more to a kind of mitochondrial dysfunction [23] caused by a supraphysiological glucose concentration.

In a glucose-free assay environment with only pyruvate and amino acids available as energy sources there were only very small differences among all four phenotypes. However, a comparison of respiratory parameters between assay media with and without glucose showed dramatically lower respiratory capacities of the two glucose phenotypes in the no-glucose environment. One possible explanation for this observation is that the available substrates in this experiment limit achievable maximal respiration. The fact that basal respiration and ATP turnover-driven respiration are virtually the same regardless of glucose availability during the assay argues against the possibility of an adverse reaction to sudden glucose withdrawal.

Finally we measured respiration of C2C12 myotubes differentiated in low glucose (1 g/l) and in high glucose (5 g/l) (Table 4). Unlike in a previous study [12], the high-glucose conditions caused the cells to maintain a significantly higher basal cellular and mitochondrial respiration with higher ATP turnover-driven respiration. However, uncoupled maximal respiration and spare respiratory capacity were higher in the low-glucose phenotype, which agrees with the results of Mailloux et al. [12] that maximum respiratory capacity was lower in cells grown and differentiated in high (25 mM) glucose. These findings suggest a higher dependence on oxidative glucose metabolism in the high-glucose phenotype under basal conditions accompanied by metabolic dysfunction under stress conditions and/or smaller spare mitochondrial respiratory machinery. It should also be noted that basal respiration of myotubes is almost twice as high as that of myoblasts under the same assay conditions, which is in good agreement with previous studies [22].

**Table 4:** Mitochondrial respiratory parameters of myotubes in glucose 1 g/l assay medium (pmol(O_2_).min^−1^.(µg protein)^−1^).

	LG	HG
Basal respiration	19.43 [16.49,22.37]	23.05 [21.31,24.79]
Basal mitochondrial respiration	12.76 [9.73,15.79]	16.34 [13.37,19.31]
ATP turnover-driven respiration	10.22 [8.31,12.13]	12.89 [11.09,14.70]
Maximum respiratory capacity	60.77 [51.37,70.16]	44.70 [27.03,62.36]
Spare respiratory capacity	48.01 [41.64,54.37]	28.36 [9.68,47.03]
H^+^ Leak	2.54 [1.38,3.70]	3.45 [2.28,4.62]
Non-mitochondrial respiration	6.67 [6.17,7.17]	6.71 [5.44,7.98]

Data are shown as means and 95% CI, n = 3.

No differences in citrate synthase activity or mitochondrial yield in myoblasts (Table 6) regardless of culture conditions suggest that observed changes in mitochondrial respiratory capacity were not due to a significant increase or decrease in mitochondrial mass. Myotubes, however, had a higher CS activity than myoblasts, which could account for their higher respiratory rate. Furthermore, the LG myotube phenotype exhibited a higher mitochondrial content than the HG phenotype, which may, in part, explain the higher spare respiratory capacity we observed.

Assessment of respiratory chain activities, however, presents a more complicated picture. In myoblasts the glucose-starved phenotypes appeared to have a higher activity across the complexes compared to cells grown in the presence of glucose, although the differences were small. This suggests that the observed differences in oxidative capacity are not linked to respiratory chain capacity in any simple way but could be complicated by changes in regulatory or transport pathways. In myotubes we found significantly higher complex I and complex III activities in the LG phenotype. The differences in complex II and complex IV activity were not statistically significant which is once again consistent with the results of Mailloux et al., who observed no differences in complex IV expression in either phenotype.

Our results show that using galactose or no glucose in the cell growth medium fails as a simple method to enhance oxidative metabolism of C2C12 cells. The effects of glucose deprivation are complex and depend, among other things, on the cell type used. For C2C12 cells our data support a recommendation to use moderate glucose concentration for cultivation and avoid high-glucose growth media. Different culture media may have a significant effect on the capacity of the mitochondrial respiratory chain but the link of such a change to the extent of oxidative metabolism of such cells remains unclear.

## Materials and Methods

All materials, chemicals and substrates were purchased from Sigma-Aldrich unless stated otherwise. All assays were used according to the manufacturer’s instructions.

### Cell Culture Conditions

C2C12 cells were obtained from Sigma-Aldrich and prior to measurements of mitochondrial respiration they were grown for seven days in the various culture media (low glucose, LG; high glucose, HG; galactose, GAL or carbohydrate-free, CF) to allow adaptation of cells. All cultures were incubated at 37°C in an atmosphere of 95% humidity and 5% CO_2_. The medium was changed every 48 hours.

All variants of growth media consisted of Dulbecco-modified Eagle’s medium (DMEM, Life Technologies) containing no glucose and supplemented with 10% fetal bovine serum, 100 unit/ml penicillin- 100 µg/ml streptomycin, and 1 mM sodium pyruvate. LG and HG medium further contained 1 g/l or 5 g/l of d-glucose respectively. GAL medium was supplemented with 1 g/l d-galactose, and CF medium was left without any carbohydrate addition.

To induce differentiation, cells were plated in culture plates or Seahorse XF24 cell plates and were allowed to grow to approximately 80–90% confluence in the growth medium. The growth medium was then replaced with a differentiation medium (DMEM with supplemented with 2% FBS, 100 unit/ml penicillin and 100 µg/ml streptomycin, 1 mM sodium pyruvate) and changed every 48 hours [12], [24] for seven days prior to respiration assay. The LG phenotype was treated with growth and differentiation media containing 1 g/l glucose, while the HG phenotype was treated with media supplemented with 5 g/l glucose.

### Measurement of Growth Rate and Doubling Time

Cells were seeded at 50,000/well in 6-well plates. Cells were then trypsinized at specified time points and counted in triplicates using a haemocytometer. The doubling time was calculated using non-linear regression.

### Galactose Assay

Samples of culture medium were taken at various time intervals and the galactose concentration was measured using AmplexRed galactose/galactose oxidase kit (Life Technologies) according to manufacturer’s instructions.

### Analysis of Metabolism

Cellular respiration was measured using the XF-24 analyser (Seahorse Bioscience). We performed mitochondrial bioenergetic assays based on published protocols [25]. The XF assay medium (HCO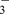 free modified DMEM, Seahorse Bioscience) was supplemented with 4 mM L-glutamine and 1 mM pyruvate and with further additions relevant to the experiment. The pH was adjusted to 7.4 at 37°C.

For myoblast experiments we seeded 25,000 cells per well of LG and HG phenotypes, and 30,000 cells per well of GAL and CF phenotypes onto analysis plates and left overnight to attach in the respective culture medium to obtain a monolayer of cells. The higher seeding number of GAL and CF cells was to compensate for slower growth rates. For myotube experiments cells were grown to confluence and differentiated for seven days in Seahorse assay plates as described above.

Mitochondrial respiration test was then performed by sequential additions of 1 µM oligomycin, 0.4 µM FCCP and 1 µM rotenone/antimycin A in the case of myoblasts and 1 µM oligomycin, 1.5 µM FCCP and 1 µM rotenone/antimycin A in the case of myotubes.

We determined the following mitochondrial parameters: basal respiration, basal mitochondrial respiration (basal cellular respiration minus non-mitochondrial respiration), ATP turnover-driven respiration (basal respiration minus oligomycin-inhibited respiration), maximal respiratory capacity (maximal uncoupled respiration minus non-mitochondrial respiration), spare respiratory capacity (maximal uncoupled respiration minus basal respiration), proton leak (oligomycin-inhibited respiration minus non-mitochondrial respiration) and non-mitochondrial respiration (rotenone/antimycin A-inhibited respiration). The results were expressed in pmol(O_2_).min^−1^.(µg protein)^−1^.

Extracellular acidification rate (ECAR) was measured in parallel with respiration and the following parameters were established: basal ECAR, oligomycin-stimulated ECAR, and glycolytic reserve (oligomycin-stimulated minus basal ECAR). The results were expressed in mpH.min^−1^.(µ g protein)^−1^.

In a parallel experiment cells were seeded identically into assay plates and then at the time of measurement lysed to quantify the protein content using QuantiPro BCA assay kit for protein determination (Sigma), which was then used to normalise the respiration data.

### Citrate Synthase Activity

Citrate synthase activity was measured in whole cell lysates using the Citrate synthase assay kit, (Sigma) in an assay mixture containing 500 µM oxaloacetate, 300 µM acetyl-CoA, 100 µM 5,5′-dithiobis(2-nitrobenzoic acid). The activity was calculated using the linear increase in absorbance at 412 nm over 1.5 minutes and normalised to protein content.

### Immunofluorescence

Immunofluorescence was carried out in dishes containing cover slides, where cells had been seeded and became adherent then differentiation was induced. Cells were rinsed with warm PBS then fixed with 4% paraformaldehyde in PBS for 30 minutes at room temperature, then washed with PBS and permeabilized with 0.1% Saponin for 30 minutes. Cover slides were carefully washed with PBS then incubated with primary monoclonal anti-myosin (skeletal, slow) antibody (Sigma) at a dilution 1∶4000 in PBS at 4°C over night. On the following day slides were carefully washed with PBS and secondary anti-mouse Alexa Fluor 488 conjugate antibody (Life Technologies), was applied at a dilution of 1∶200 in PBS for one hour. Final rinsing with PBS and cover slides were mounted using Prolong Gold Antifade Reagent (Life Technologies). Pictures were taken using Leica TCS SP II Confocal microscope.

### Preparation of Enriched Mitochondrial Fractions

Cells were harvested and centrifuged in a swinging-bucket rotor at 1000×g room temperature for 15 minutes. Supernatant was removed and the pellets were suspended in 750 µl ice-cold cell homogenization medium (10 mM Tris-Cl, 10 mM KCl, 150 µM MgCl_2_, pH 6.7). We disrupted the cells mechanically with a 2 ml glass tight-fitting Dounce homogenizer (Wheaton type B) on ice. After homogenization we added 250 µl ice-cold cell homogenization medium supplemented with 1 M sucrose and centrifuged the homogenate (5 min at 1000×g and 4°C) in a fixed-angle rotor. The 1 ml supernatant was removed and centrifuged again (10 min at 5000×g and 4°C), and the resulting supernatant was carefully removed. The mitochondrial pellet was resuspended in 0.5 ml of sucrose/Mg^2+^ medium (10 mM Tris-Cl, 150 µM MgCl_2_, 250 µM sucrose, pH 6.7) and centrifuged (10 min at 5000×g and 4°C). The pellet was resuspended in mitochondrial separation medium (10 mM Tris-Cl, 250 µM sucrose, pH 7.0) then frozen and kept at −80°C.

### Spectrophotometric Mitochondrial Respiratory Chain Activity Assays

We measured complex I and complex II activities as described in [26]. Complex I assay was performed in an assay mixture composed of 20 mM potassium phosphate, 3.5 g/l BSA, 60 µM 2,6-dichloroindophenol (DCIP), 70 µM decylubiquinone, 1 µM antimycin A and 0.2 mM NADH, pH 7.4. Complex II activity was measured in an assay mixture containing 80 mM potassium phosphate, 1 g/l BSA, 2 mM EDTA, 10 mM succinate, 1 mM sodium azide, 80 µM DCIP, 50 µM decylubiquinone, 1 µM antimycin A and 3 µM rotenone, pH 7.8. Complex III activity was measured as described in [27] in an assay mixure containing 50 µM cytochrome *c*, 50 mM Tris-Cl, 4 mM sodium azide and 50 µM decylubiquinol, pH 7.4. Decylubiquinol was prepared by dissolving decylubiquinone in acidified ethanol pH 4 then reduced by adding few grains of sodium borohydride NaBH_4_ and vortexed till the solution became colourless then aliquots were stored at −20°C. Complex IV activity was measured as described in [28] in assay buffer containing 30 mM potassium phosphate and 50 µM of freshly reduced cytochrome *c*, pH 7.4. All assays were modified for use in a microplate reader.

### Statistical Analyses

Data are presented as mean or difference of means and 95% confidence interval (CI) [29], [30]. We performed one-way ANOVA with Tukey post-hoc test for statistical significance of difference of means or t-test with alpha set to 0.05. Differences passing these tests are marked with an asterisk in the text. Statistical analyses were performed using GraphPad Prism version 5.0 d for Mac OS X, GraphPad Software, San Diego California USA, www.graphpad.com.
